# TgDDP is a novel DIX domain protein that plays an essential role in *Toxoplasma* endodyogeny

**DOI:** 10.1128/mbio.01952-26

**Published:** 2026-09-28

**Authors:** Rajshekhar Y. Gaji, Padmaja Mandadi, Dima Ali, Isabelle Coppens, Foster K. Agyei, Birgit E. Scharf, Sangita Karki, Frank Aylward, William J. Hanrahan, Anne M. Brown

**Affiliations:** 1 Department of Biomedical Sciences and Pathobiology, Virginia-Maryland College of Veterinary Medicine 229659, Blacksburg, Virginia, USA; 2 Department of Molecular Microbiology and Immunology, Johns Hopkins Bloomberg School of Public Health 25802 , Baltimore, Maryland, USA; 3 Department of Biological Sciences, Virginia Tech 1757, Blacksburg, Virginia, USA; 4 Department of Biochemistry, Virginia Tech 1757, Blacksburg, Virginia, USA; UT Southwestern Medical Center, Dallas, Texas, USA

**Keywords:** apicomplexan parasites, *Toxoplasma gondii*, cell division, protozoa

## Abstract

Apicomplexan parasites are protozoan pathogens responsible for major human diseases, including toxoplasmosis, malaria, and cryptosporidiosis. *Toxoplasma gondii* has emerged as a model for studying cell division in tissue coccidians. Unlike higher eukaryotes that divide by binary fission, *Toxoplasma* replicates via internal budding (endodyogeny), yet many factors governing daughter cell biogenesis remain poorly understood. Here, we characterize TgDDP (*T. gondii* DIX–DnaJ domain protein), a previously unannotated protein containing both DIX and DnaJ domains—a unique combination restricted to stramenopiles and apicomplexans. Endogenous tagging revealed that DDP is constitutively expressed and localizes predominantly to the trans-Golgi network in extracellular parasites, with a more dispersed distribution in intravacuolar stages. To assess function, we generated a conditional knockdown line using the auxin-inducible degron system. DDP depletion resulted in a complete block in parasite replication, demonstrating its essential role in the lytic cycle. Microscopy analyses showed that loss of DDP disrupts daughter cell scaffold formation, while nuclear division remains largely intact, indicating a specific role after karyokinesis. Complementation with a wild-type TgDDP allele fully restored parasite replication, and orthologs from related apicomplexans functionally substituted for DDP. Biochemical analysis (SEC–MALS) demonstrated that DDP forms dimers, consistent with DIX domain-containing proteins. Interactome profiling further identified associations with trafficking-related proteins, including clathrins. Together, these findings establish DDP as a critical regulator of daughter cell formation and a potential therapeutic target to inhibit parasite propagation.

**IMPORTANCE:**

*Toxoplasma gondii* is a protozoan parasite that can cause life-threatening disease in humans with immunodeficiency conditions; hence, identifying key factors required for parasite propagation is important to develop novel therapeutics. In this study, we characterize a novel DIX domain-containing protein, DDP, expressed in *Toxoplasma*. We show that DDP mostly localizes to the trans-Golgi network in extracellular stages and exhibits dispersed localization in the intracellular form. To determine the function of DDP, we generated a conditional knockdown strain and showed that loss of this protein results in abrogation of parasite division. Specifically, parasites lacking DDP are unable to form daughter parasites. Further, the DDP interactome includes trafficking proteins clathrins, along with IMC proteins, thus suggesting that DDP plays an essential role in building daughter cells during endodyogeny.

## INTRODUCTION

Cell division is a fundamental biological process essential for growth, development, and reproduction across all living organisms. Despite its universality, the mechanisms and strategies of cell division can vary dramatically among different lineages, reflecting adaptation to distinct cellular architectures and ecological niches. Among eukaryotes, protists represent a remarkably diverse group in which cell division often exhibits unique and highly specialized features. The SAR (Stramenopiles, Alveolates, and Rhizaria) supergroup is a major subdivision of protists, comprising three large lineages of plastid-containing unicellular organisms that display a wide range of cellular and reproductive strategies (1, 2). Within this group, the Alveolata include apicomplexan parasites, which are responsible for significant human and animal diseases (1).

Cell division in apicomplexans is exceptionally complex and shows remarkable variation across species (3). Nevertheless, recent studies have suggested that this process can be broadly deconstructed into four modular events: disassembly of the mother’s cytoskeleton, DNA replication and segregation, nuclear division, and daughter cell budding. While these modules are conserved, their temporal sequence may differ between species (4). Much of our current understanding of apicomplexan cell division has been derived from studies on *Toxoplasma*, a model parasite amenable to diverse genetic manipulations (5–7).

*Toxoplasma gondii* is a significant human pathogen responsible for potentially fatal encephalitis in immunodeficient individuals, miscarriage in pregnant women, and blindness or cognitive impairment in congenitally infected children (8–10). As an obligate intracellular parasite, *Toxoplasma* can replicate only within a host cell. Its replication follows a unique mode called endodyogeny (11–13), which differs markedly from mammalian cell division. In metazoans, cell division involves “open mitosis,” during which the nuclear envelope breaks down to allow the mitotic spindle to form, followed immediately by cytokinesis to produce two daughter cells. By contrast, *Toxoplasma* undergoes “semi-closed mitosis,” in which the nuclear envelope remains largely intact throughout division (14). Furthermore, cytokinesis in *Toxoplasma* begins before mitosis is complete and proceeds through internal budding of two daughter parasites identical to the mother. Consequently, unlike mammalian cells, daughter cells in *Toxoplasma* do not result from fission of the mother cell but are assembled within it (3, 4). Understanding the mechanisms of endodyogeny and identifying its key components could therefore provide avenues for developing selective strategies to disrupt parasite replication and infection.

The process of endodyogeny in *Toxoplasma* begins with the duplication of the Golgi apparatus late in G1 and centrosome division early in S phase (13, 15–19). Budding is initiated late in S phase before the onset of mitosis when the initial components of the cytoskeleton, including inner membrane complex (IMC) components, begin to assemble apical to the recently duplicated centrosomes (13, 17, 20, 21). Mitosis and cytokinesis progress simultaneously as the cytoskeleton extends from the apical end toward the basal region, encapsulating first the divided Golgi, followed by the apicoplast, nucleus, endoplasmic reticulum (ER), and finally the mitochondrion (13, 19, 21, 22). The secretory organelles, including micronemes, rhoptries, and dense granules, are formed *de novo* (19). As the daughter parasites reach maturity, the cytoskeleton of the mother disintegrates, and the plasma membrane of the mother is recycled onto the emerging daughters along with newly generated membrane (5, 13).

Although recent studies have made significant progress in identifying protein components involved in the formation of daughter cell organelles during endodyogeny, very few proteins associated with the biogenesis of daughter cell IMCs have been characterized. Therefore, identification and functional characterization of new proteins involved in trafficking and assembly of progeny IMCs would not only address critical gaps in understanding *Toxoplasma* division but also potentially inform the development of novel therapeutic strategies.

In this study, we investigated a novel protein, TgDDP (*Toxoplasma gondii* DIX and DnaJ domain-containing protein), and demonstrated that it is essential for *Toxoplasma* division. Specifically, we show that DDP predominantly localizes to the trans-Golgi network within the parasite and that loss of this protein results in a complete disruption of daughter cell budding and overall cell division during endodyogeny.

## RESULTS

### Proteins containing both DIX and DnaJ domains are restricted to stramenopiles and apicomplexans

DIX domain–containing proteins are widespread across both plant and animal kingdoms (23). In animals, DIX domain proteins such as AXIN play a key role in the Wingless-related integration site (Wnt)–β-catenin signaling pathway, which regulates embryonic development (24). In plants, recent studies have demonstrated that SOSEKI proteins containing DIX domains are involved in the establishment of cell polarity (23). Previous reports suggest that DIX domain–containing proteins are conserved in both unicellular and multicellular organisms (23, 25, 26). However, little is known about the presence or function of DIX family proteins in the protozoan parasite *Toxoplasma gondii*.

To determine whether DIX domain–containing proteins are present in *Toxoplasma*, we performed a bioinformatic analysis of the parasite genome. This analysis identified two proteins containing DIX domains. TGGT1_220090 contains only a DIX domain, whereas TGGT1_261410 contains both a DIX domain and a DnaJ domain. We designated TGGT1_220090 as TgDCP (*Toxoplasma gondii* DIX domain-containing protein) and TGGT1_261410 as TgDDP (*Toxoplasma gondii* DIX–DnaJ domain protein).

**Fig 1:**
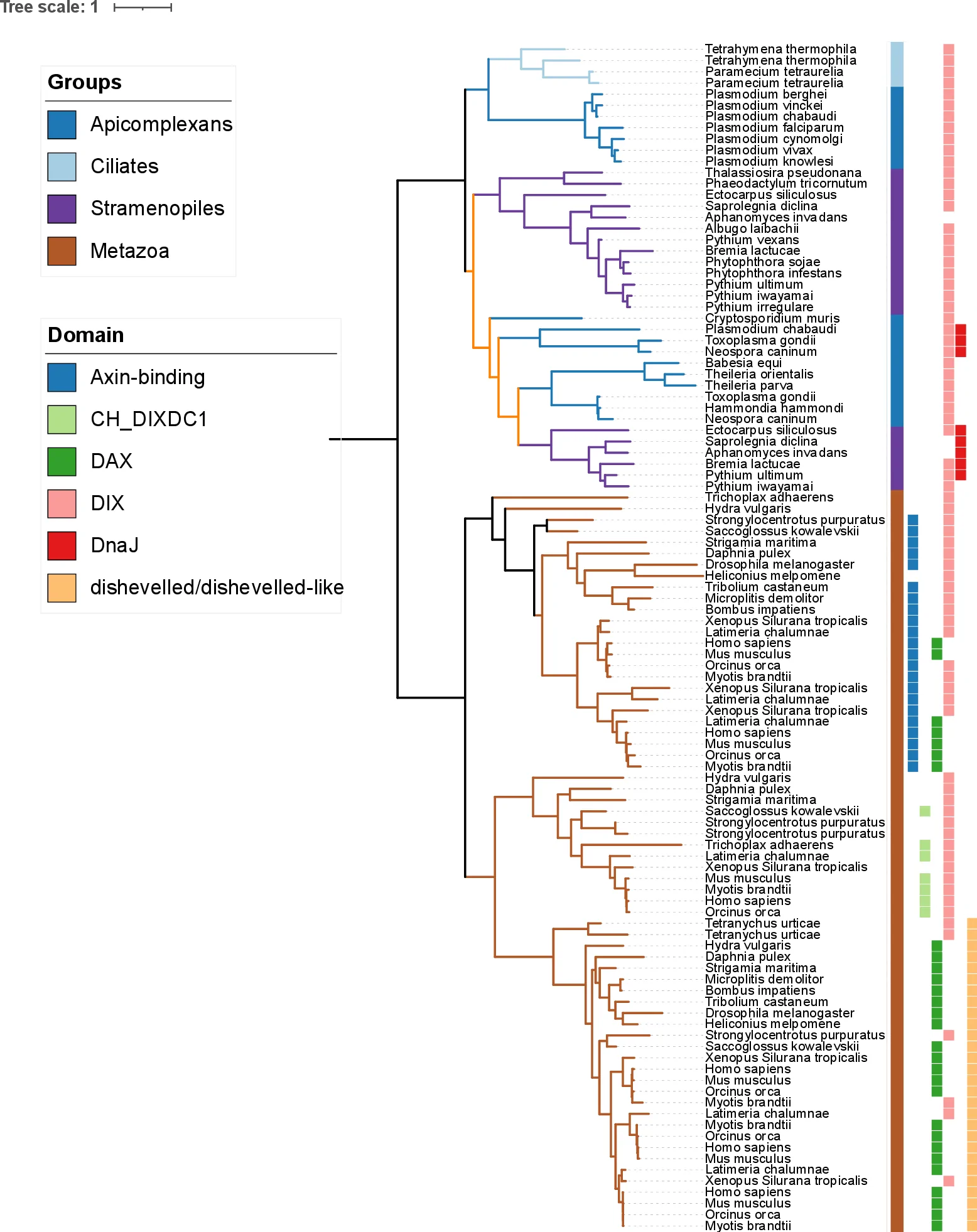
Proteins with both DIX and DnaJ are confined to Stramenopiles and Apicomplexans. Phylogenetic tree based on the DIX domain protein for different eukaryotic groups was inferred using the JTT + F + R6 model, which was chosen as the best-fit model by the ModelFinder utility in IQ-TREE. The inner strip represents the different taxonomic groups within eukaryotes, while the outer strip represents the presence or absence of Pfam domains associated with the DIX domain proteins.

The DnaJ domain is characteristic of the heat shock protein 40 (Hsp40) family of molecular chaperones, which modulate the activity of Hsp70 family proteins. Together, Hsp40 and Hsp70 play essential roles in diverse cellular processes, including protein folding and intracellular trafficking. Phylogenetic analysis of DIX and related domains (Disheveled, DAX, CH_DIXDC1, and AXIN-binding proteins) across eukaryotes revealed that, although DIX-related domain-containing proteins and DnaJ domain-containing proteins are common in higher eukaryotes, proteins harboring both domains within a single polypeptide are restricted to stramenopiles and apicomplexans (26) (Fig. 1).

Given that TGGT1_261410 represents a unique fusion of DIX and DnaJ domains within a single protein, we focused our subsequent analyses on elucidating the role of this novel protein in *Toxoplasma* biology.

### DDP predominantly localizes to the trans-Golgi network (TGN)

**Fig 2:**
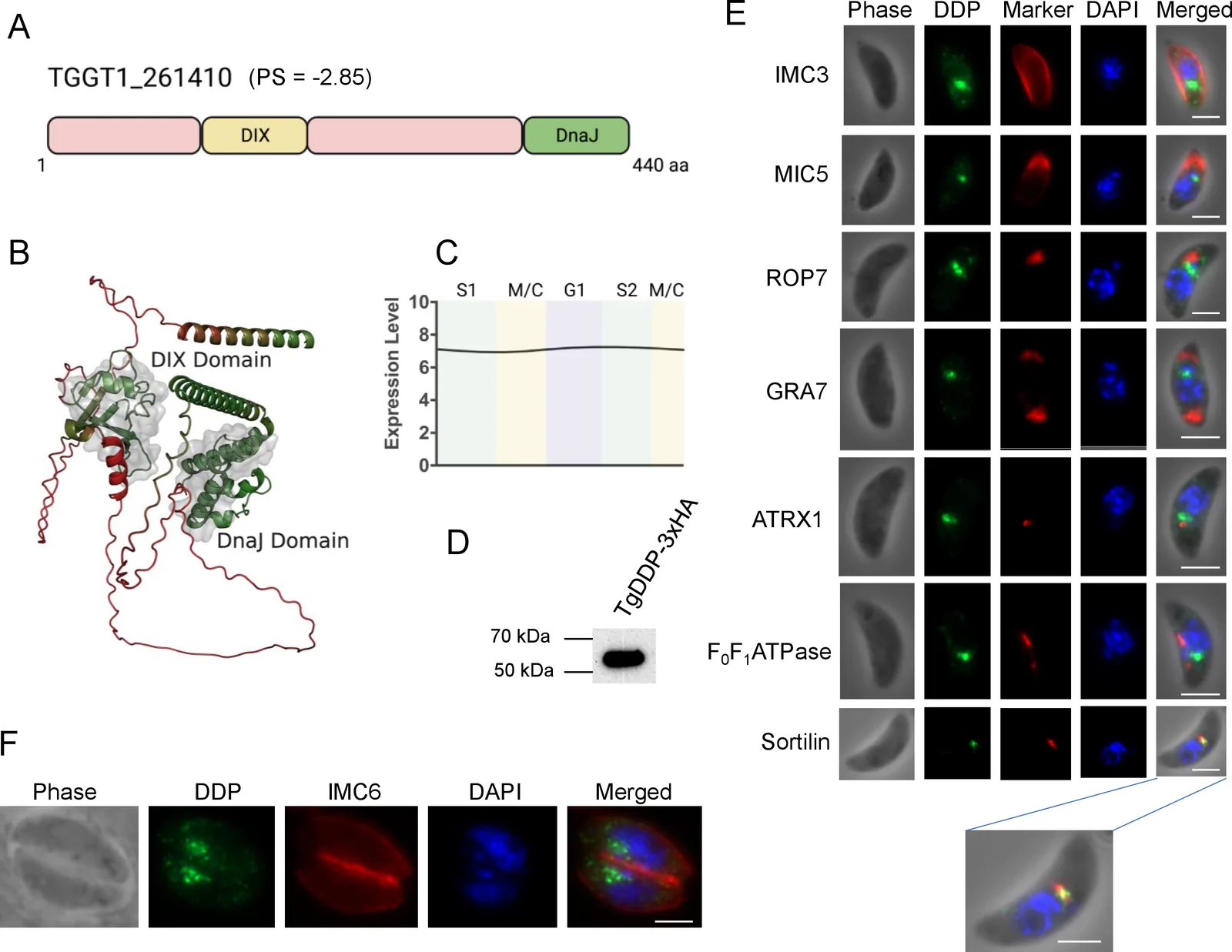
TgDDP shows predominant localization to the trans-Golgi network in *Toxoplasma*. (**A**) Schematic representation of TgDDP protein. The protein is 440 amino acids in length. The location of DIX and DnaJ domains is indicated. CRISPR screen phenotype score for TgDDP is also shown. (**B**) TgDDP AlphaFold3 model showing DIX domain (residues 120–179) and DnaJ domain (residues 394–437) highlighted in gray and labeled accordingly. The coloring plots pLDDT data onto the structure, where green represents increased confidence and red indicates decreased confidence. (**C**) Cell cycle expression profile of TgDDP protein in tachyzoite stage from previously published study (30). The expression pattern indicates that TgDDP is constitutively expressed throughout the cell cycle. (**D**) Immunoblot analysis of TgDDP.3×HA strain using anti-HA antibody. (**E**) IFA analysis of extracellular parasites using anti-HA antibody and antibodies against different organellar proteins. DDP is shown in green, organellar marker in red, and DAPI (blue) stains the nucleus. (**F**) Localization of TgDDP in intracellular parasites. IMC6 stains the inner membrane complex, and DAPI stains the nucleus. Scale bar: 2 μm.

The protein TgDDP (hereafter referred to as DDP) is 440 amino acids in length, with the DIX domain toward the N-terminus and a DnaJ domain at the C-terminal region (Fig. 2A). AlphaFold3 (27) modeling of DDP revealed that the DIX domain (residues 120–179) is made of one α-helix and two β-sheets. The DnaJ domain (residues 394–437), however, consists of two α-helices (Fig. 2B). pLDDT confidence values indicate that both the DIX and DnaJ domains correspond to regions of relatively high structural confidence, while much of the remaining sequence is designated as low confidence and thus predicted to be intrinsically disordered regions. Independent secondary structure predictions from Psipred (28, 29) show strong agreement with the AlphaFold3 model for these domains, further supporting the predicted fold. Together, these analyses indicate that the DIX and DnaJ domains represent the most structurally reliable regions of DDP, supporting their use for subsequent structural and functional interpretation.

A previous transcriptomic analysis of genes expressed during the tachyzoite stage showed that DDP is constitutively expressed in *Toxoplasma* (30) (Fig. 2C). To determine the subcellular localization of DDP, we endogenously tagged the *TgDDP* gene with a C-terminal 3×HA epitope using CRISPR/Cas9-mediated genome editing (31, 32). Immunoblot analysis using an anti-HA antibody detected a single band corresponding to the predicted molecular weight of DDP (Fig. 2D). Immunofluorescence analysis (IFA) revealed weak punctate cytoplasmic staining and a strong signal in the region between the apicoplast and the nucleus in extracellular parasites (Fig. 2E).

To precisely identify the compartment in which the DDP shows a strong signal, we used markers for different organelles including IMC, micronemes, rhoptry, dense granules, apicoplast, mitochondrion, and trans-Golgi network in extracellular stages. Interestingly, DDP showed colocalization with the trans-Golgi network marker Sortilin (33) (Fig. 2E), suggesting that the protein predominantly localizes to the parasite trans-Golgi network. In intracellular parasites, DDP displayed a slightly more diffuse localization pattern in the region between the nucleus and the apicoplast, along with punctate cytosolic staining (Fig. 2F).

### DDP exhibits dynamic localization during cell division in *Toxoplasma*

To examine DDP localization during *Toxoplasma* endodyogeny, we performed an immunofluorescence assay (IFA) on intracellular parasites at different stages of cell division. DDP exhibited dynamic localization patterns throughout the parasite cell cycle, appearing more compact during the G1 phase and becoming progressively more diffuse as the cell cycle advanced. Notably, a fraction of DDP colocalized with the inner membrane complex (IMC) of nascent daughter parasites during the mitosis/cytokinesis (M/C) stages of parasite division (Fig. S1A). Quantitative analysis revealed that 83% of vacuoles during the G1 phase exhibited a compact DDP staining pattern, whereas 67% of vacuoles in the early M/C phase showed diffuse DDP localization. During the late M/C phase, 69% of vacuoles exhibited DDP association with the IMC (Fig. S1B).

### DDP is essential for *Toxoplasma* propagation

**Fig 3:**
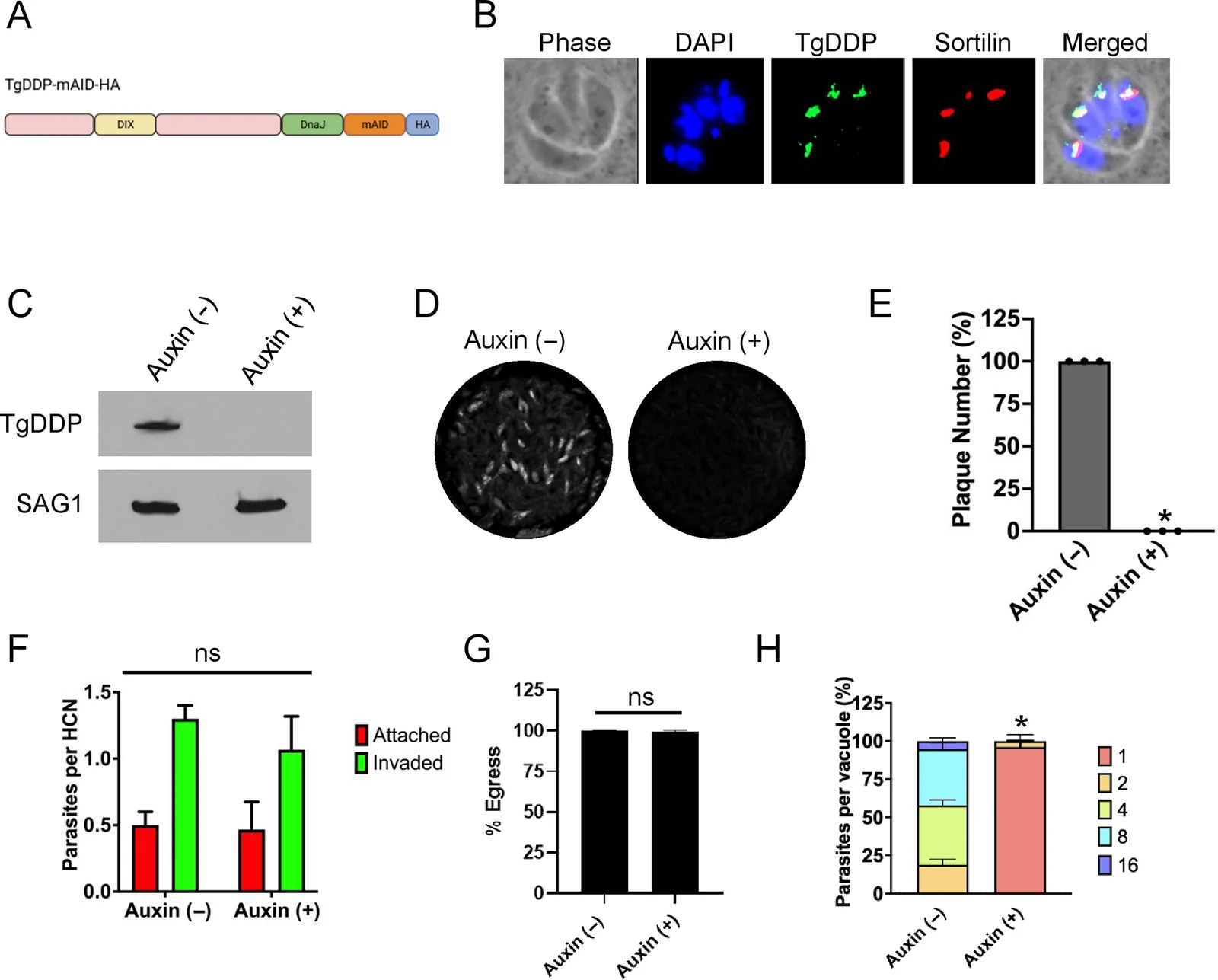
TgDDP is essential for *Toxoplasma* endodyogeny. (**A**) Schematic representation of mAID.3×HA tagged TgDDP. (**B**) IFA showing localization of mAID.3×HA tagged TgDDP protein in intracellular parasites. DDP is shown in green, Sortilin in red, and DAPI (blue) stains the nucleus. (**C**) Immunoblot analysis of TgDDP.mAID.3×HA with anti-HA antibody in the presence or absence of auxin. SAG1 is used as a loading marker. (**D**) Plaque assay of TgDDP.mAID.3×HA in the presence or absence of auxin. (**E**) Quantification of plaque numbers. *n* = 3, error bars = SD, Student’s *t*-test. *P < 0.05. (**F**) Host-cell invasion assay of freshly egressed TgDDP.mAID.3×HA parasites after 4 h of vehicle control or auxin treatment. Data are compiled from three independent experiments, and error bars represent standard deviations. ns, not significant, Student’s *t*-test. (**G**) Ionophore-induced egress assay of intracellular TgDDP.mAID.3×HA parasites following 4 h of auxin or vehicle treatment. Data are compiled from three independent experiments, and error bars represent standard deviations. ns, not significant, Student’s *t*-test. (**H**) Replication assay of TgDDP.mAID.3×HA in the presence or absence of auxin. *n* = 3, error bars = SD, Student’s *t*-test. *P < 0.05.

Next, we wanted to determine the role of DDP in the lytic cycle biology of *Toxoplasma*. A previous genome-wide screen aimed at identifying genes important for parasite fitness suggested that *TgDDP* is an essential gene in *Toxoplasma* (34). Since *TgDDP* is predicted to be an essential gene, we decided to generate a conditional knockdown DDP to understand its role in *Toxoplasma* biology. To achieve this objective, we inserted an auxin-inducible degron (mAID)−3×HA tag at the C terminus through CRISPR-Cas9 technology (35) (Fig. 3A). We verified the expression of mAID.3×HA and the correct localization of tagged DDP using IFA (Fig. 3B). Importantly, when auxin is added to the TgDDP.mAID.3×HA strain, the DDP is degraded within 2 h, suggesting that our conditional knockdown system is working (Fig. 3C).

With this strain in hand, we next examined the effect of the loss of DDP on parasite growth *in vitro* by monitoring the formation of plaques on a confluent human foreskin fibroblast (HFF) monolayer that results from multiple rounds of the lytic cycle (5, 6). We observed that while the TgDDP.mAID.3×HA strain treated with vehicle control formed plaques as expected, the auxin-treated parasites did not form any plaques, suggesting that DDP is essential for *Toxoplasma* growth *in vitro* (Fig. 3D and E).

### DDP is essential for *Toxoplasma* replication

An impairment of plaque formation can be caused by defects in one or more steps of the parasite lytic cycle (6, 36). Therefore, we next sought to determine which aspect of the lytic cycle was impaired in the absence of DDP protein. Toward this goal, we performed parasite invasion assays into host cells (37) and ionophore-induced egress assays using the Ca^2+^ ionophore A23187 (38, 39). However, we did not see statistically significant differences either in host-cell invasion or egress capability of parasites lacking DDP (Fig. 3F and G), suggesting that DDP does not play a role in *Toxoplasma* invasion or egress from the host cells.

We next assessed parasite replication using standard doubling assays (40), and 24-h post-infection, we observed that parasites treated with vehicle control showed normal growth, with a large number of vacuoles having four or eight parasites per vacuole. However, with parasites treated with auxin, we found about 98% of the vacuoles contained just a single parasite, suggesting a block in replication (Fig. 3H). These findings indicate that DDP is essential for *Toxoplasma* division and lack of this protein disrupts endodyogeny.

### DDP is essential for initiation of daughter cell budding during *Toxoplasma* endodyogeny

Since we observed that in the absence of DDP *Toxoplasma* endodyogeny is abrogated, we wanted to determine which stages of *Toxoplasma* division are affected by the loss of DDP. Toward this objective, we allowed the TgDDP.mAID.3×HA strain to invade host cells and then treated intracellular parasites with vehicle control or with auxin. We then performed IFA of intracellular parasites to determine their replication status.

**Fig 4:**
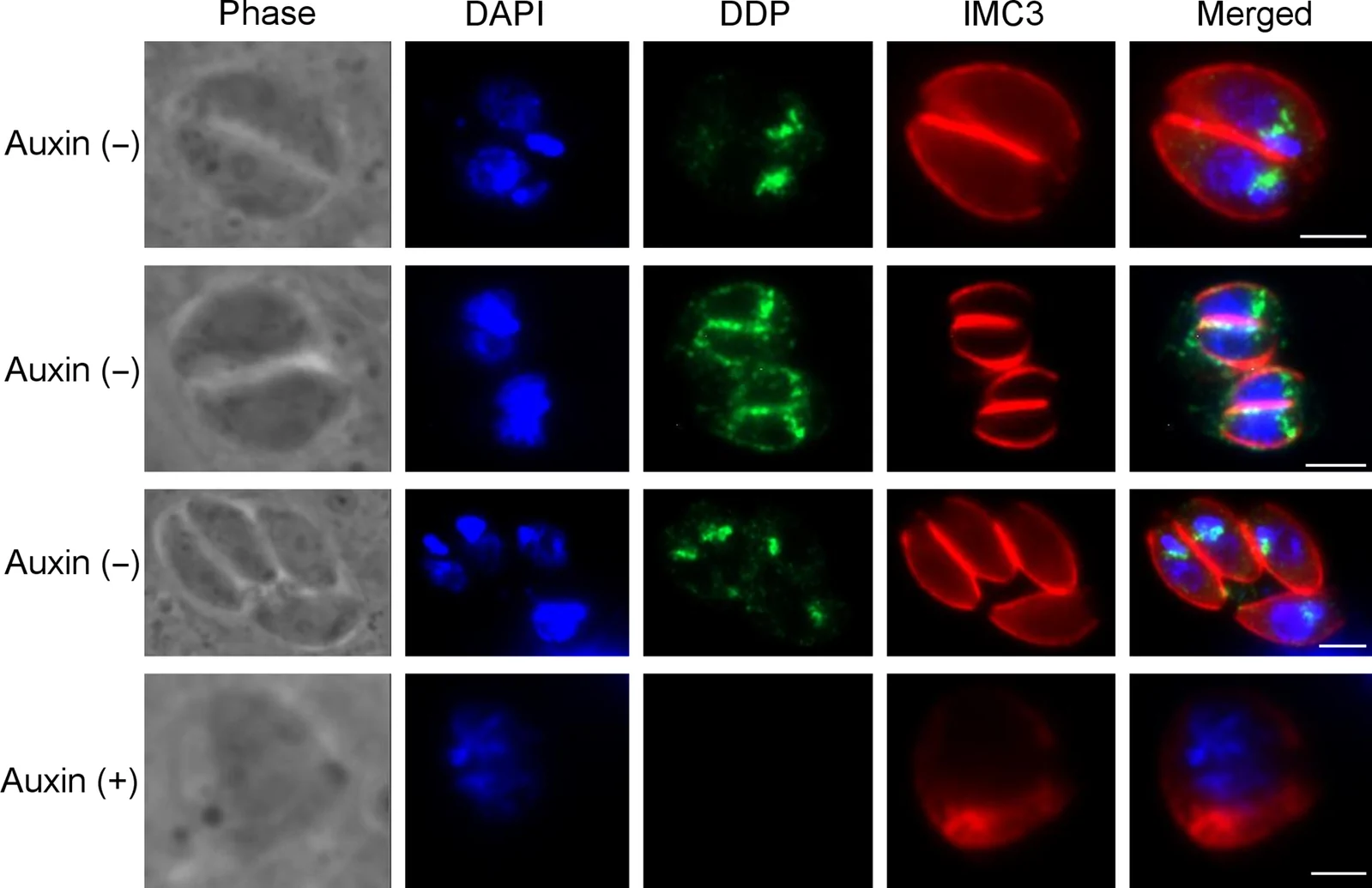
Loss of TgDDP leads to blockage of *Toxoplasma* endodyogeny. IFA analysis of TgDDP.mAID.3×HA strain in the absence (top three panels) or presence of auxin (bottom panel) using antibodies against HA and IMC3. DDP is shown in green, IMC3 in red, and DAPI (blue) stains the nucleus. Scale bar: 2 μm.

In parasites without auxin treatment, we observed actively dividing parasites at two or four parasites per vacuole, along with many in intermediate division stages, including initiation and extension of daughter buds, revealed by IMC staining (13) (Fig. 4). However, in parasites treated with auxin, the parasite morphology appeared rounded, and we did not see any vacuoles with two distinct daughter cells. Interestingly, we noticed that auxin-treated parasites were able to complete the replication of their nucleus, but we did not see initiation of daughter cell budding or cytokinesis in any of the vacuoles (Fig. 4). These results indicate that DDP is essential for daughter cell budding and cytokinesis during *Toxoplasma* endodyogeny.

### DDP depletion disrupts organelle organization and daughter cell formation

**Fig 5:**
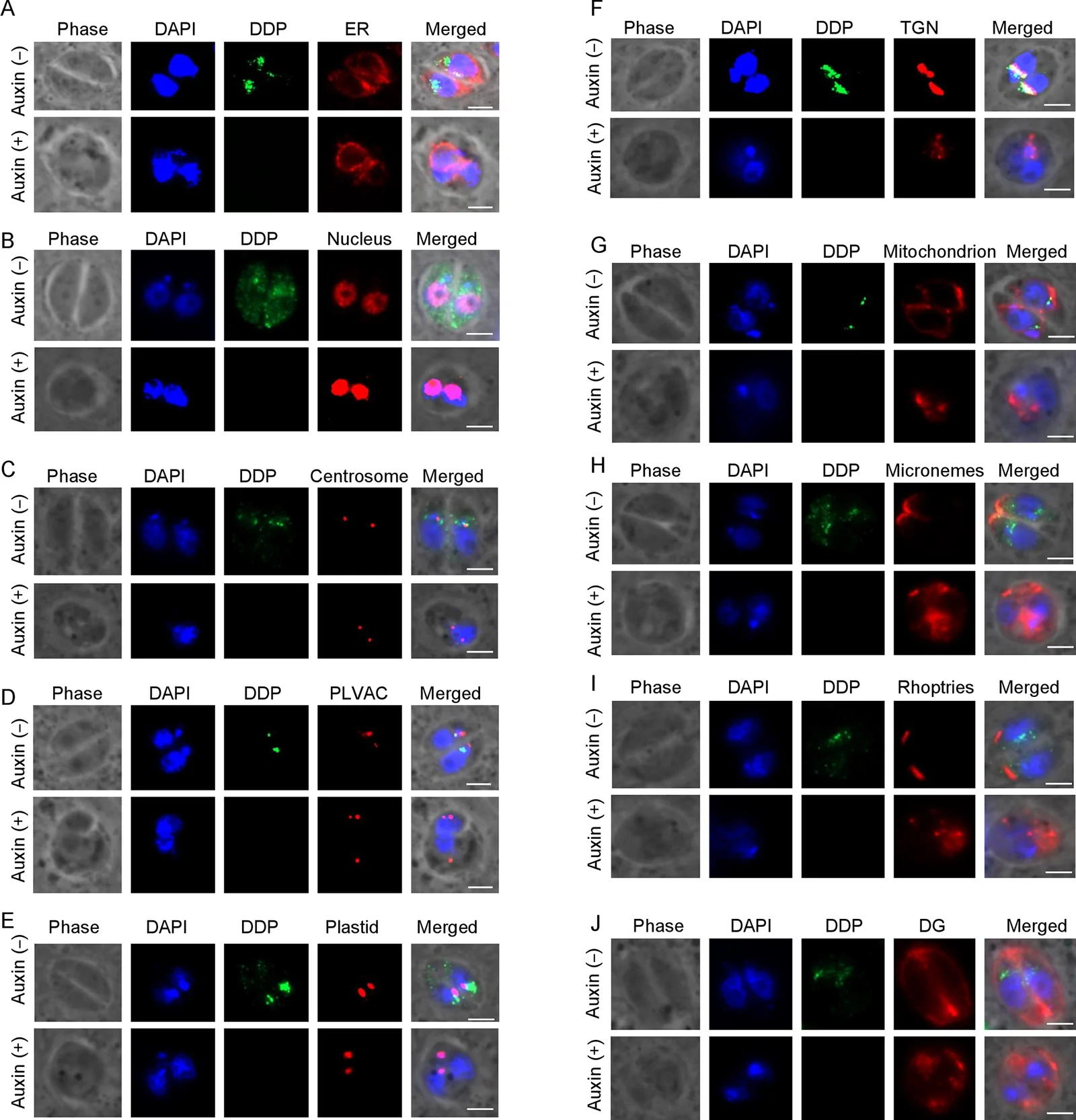
IFA analysis of TgDDP.mAID.3×HA strain in the presence or absence of auxin using antibodies against HA epitope and different organellar proteins. The different markers tested include (**A**) TgSERCA as a marker for parasite ER, (**B**) histone protein H3 as a marker for nuclear proteins, (**C**) Centrin 1 as a marker for the centrosome, (**D**) VP1 as a marker for PLVAC, (**E**) ATRX1 as a marker for the apicoplast, (**F**) Sortilin as a marker for the trans-Golgi network, (**G**) F0F1 ATPase as a marker for the mitochondrion, (**H**) M2AP as a marker for the micronemes, (I) ROP7 as a marker for rhoptries, and (**J**) GRA7 as a marker for dense granules (DG). Scale bar: 2 μm.

Since our findings revealed that lack of DDP leads to blockage of new daughter IMCs and cytokinesis, we wanted to know the replication status of different organelles in the parasite. We treated intracellular parasites with either vehicle control or auxin and performed IFA analysis using antibodies against different parasite organellar markers, including ER, nucleus, centrosome, TGN, plant-like vacuolar compartment (PLVAC), apicoplast, mitochondrion, micronemes, rhoptries, and dense granules (Fig. 5).

We observed that ER duplication occurs in DDP-deficient parasites despite the failure to complete cytokinesis and daughter cell separation (Fig. 5A). Using histone H3 as an additional nuclear marker, we found that wild-type parasites exhibited duplicated nuclei that were appropriately partitioned into newly formed daughter cells. In contrast, DDP-deficient parasites contained duplicated nuclei within a rounded, undivided parasite mass, consistent with a failure in daughter cell formation (Fig. 5B). Similarly, we saw that duplication of centrosome, PLVAC, and the apicoplast occurred in the absence of DDP, with the parasites lacking any architecture of newly formed daughter cells (Fig. 5C to E). However, the TGN compartment and mitochondrion showed fragmented appearance in the absence of DDP (Fig. 5F and G). Likewise, we also observed that proteins of the secretory organelles, including micronemes, rhoptries, and dense granules, were present in the round parasite structure in the auxin-treated parasites. However, since there is no cytokinesis, the precise localization of the secretory proteins is difficult to ascertain in parasites lacking DDP (Fig. 5H to J). Together, these findings indicate that although several organelles continue to duplicate in the absence of DDP, their proper organization and partitioning into daughter cells are disrupted, ultimately preventing the formation of clearly defined daughter cells during endodyogeny.

### DDP knockdown mutants fail to partition organelles among progeny

To further understand events during cell division in *DDP* conditional mutant, we performed electron microscopy (EM) of intracellular parasites treated with vehicle control or auxin. Specifically, we conducted EM observations to scrutinize the ultrastructure of TgDDP.mAID.3×HA parasites at two time points of infection: 12 h, corresponding to first cycle of endodyogeny, and 18 h, comprising two cycles of division.

**Fig 6:**
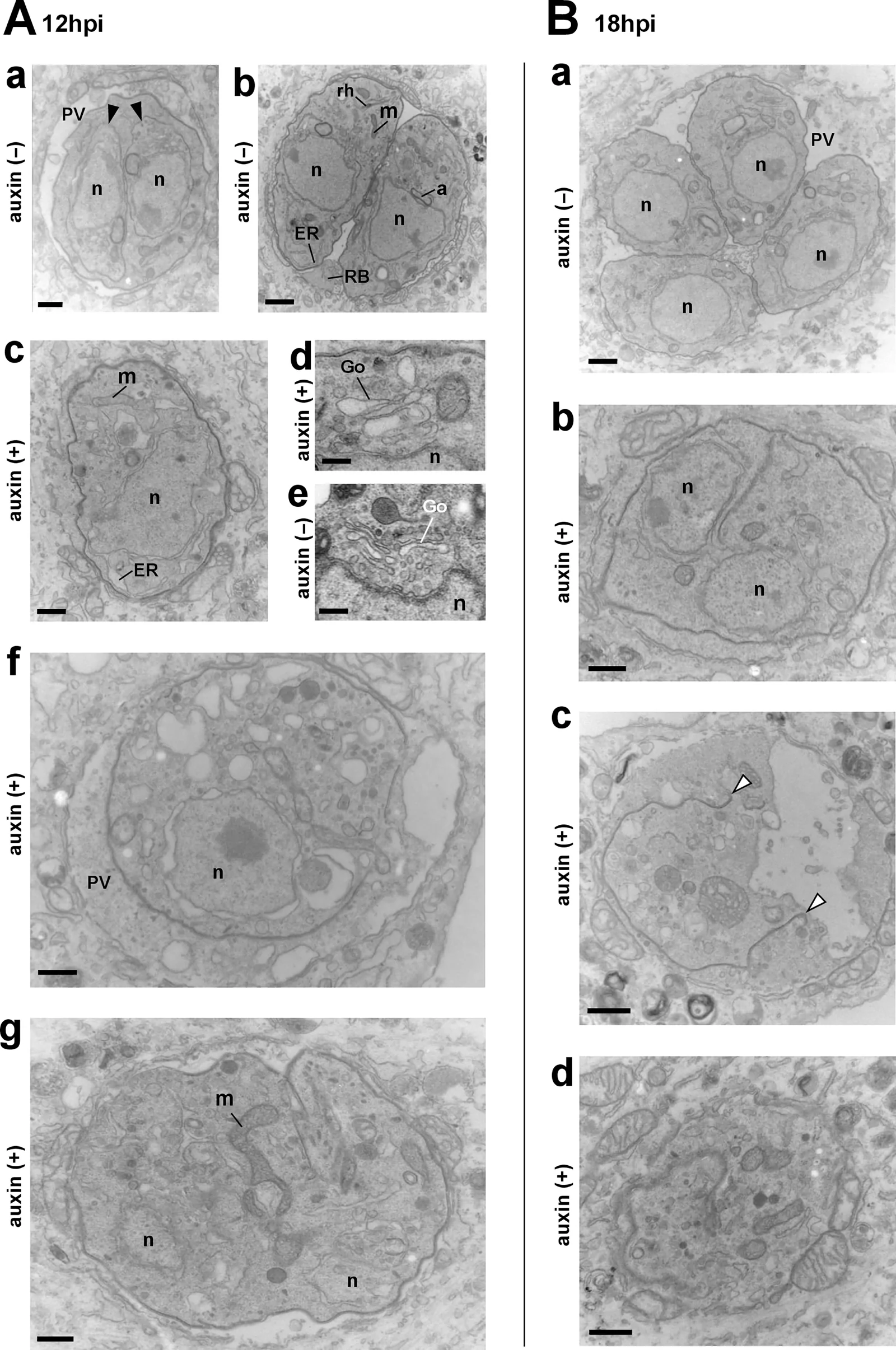
Ultrastructure of TgDDP-deficient parasites. (**A and B**) EM of wild-type and TgDDP-deficient parasites infecting HFFs at 12 h post-infection (p.i.) and 18 hpi in the presence of auxin compared to untreated *Toxoplasma*. Representative images from 19 to 46 PV are shown for the four experimental conditions, showing aberrant and incomplete endodyogeny for auxin-treated parasites. Black arrowheads show proper formation of daughters within the mother cell in untreated parasites. White arrowheads indicate points of pellicle rupture, a, apicoplast; ER, endoplasmic reticulum; Go, Golgi apparatus; IMC, inner membrane complex; m, mitochondrion; n, nucleus; RB, residual body; rh, rhoptry. Scale bars are 500 nm, except for panels d and e, corresponding to 100 nm.

In vehicle control-treated parasites, active endodyogeny at 12 h post-infection (p.i.) was evidenced by the profiles of two daughter cells developing within the confines of a mother cell (Fig. 6A, panel a), which will be consumed and then packaged into a vestigial “residual body”; the end of endodyogeny was marked by the synchronous emergence of two individualized daughters (Fig. 6A, panel b). An important hallmark of DDP knockdown parasites was the swelling of the nuclear envelope and the secretory pathway, including the ER and TGN (Fig. 6A, panels c and d), compared to untreated parasites (Fig. 6A, panels b and e).

We also observed extensive vacuolization, suggesting that loss of DDP may impose cellular stress on the parasite (Fig. 6A, panel f). Subsequently, treated parasites lost their polarity and became roundish. Despite these striking anomalies, DDP-deficient parasites could undergo karyokinesis, as illustrated by the detection of two nuclear profiles in the cytoplasm (Fig. 6A, panel g). However, fully formed progeny was never observed, indicative of abortive endodyogeny (Fig. 6A, panel g). At 18 h p.i., the majority of control parasitophorous vacuoles (PV) contained four parasites (Fig. 6B, panel a). In contrast, by that time, no DDP-deficient parasites could undergo cytokinesis after the karyokinesis (Fig. 6B, panel b). In their failed attempt to partition organelles among progeny, these parasites were composed of a mix of maternal and daughter organelles, resulting in plasmalemma breakdown (arrowheads in Fig. 6B, panel c) and eventually death due to leakage of the cellular contents into the PV (Fig. 6B, panels c and d).

### Complementation of DDP conditional mutant with wild-type copy of DDP overcomes cell division defect

Next, we wanted to determine if the cell division defect seen with lack of DDP in the TgDDP.mAID.3×HA strain can be overcome by introducing a wild-type copy of DDP. Toward this goal, we directed a DNA fragment of *TgDDP*-cDNA under the control of the native promoter into the *UPRT* locus through CRISPR-Cas9 technology. To verify that the extra copy of *DDP* is functional, we performed plaque assays by treating parental (TgDDP.mAID.3×HA) and TgDDP-WT-COMP with either vehicle control or auxin. The results revealed that the parental clone was able to grow in the absence of auxin but did not form any plaques in the presence of auxin, as expected (Fig. S2A and B). However, the complemented strain was able to form plaques both in the absence or presence of auxin, suggesting that the extra copy of *DDP* is functional and hence is able to successfully rescue the phenotype (Fig. S2C and D). We also performed immunofluorescence assays, and the findings revealed that normal endodyogeny was indeed restored in the TgDDP-WT-COMP strain (Fig. S2E).

### TgDDP orthologs from related apicomplexans rescue the phenotype of the DDP conditional knockdown mutant

TgDDP belongs to a class of DIX-DnaJ proteins found in SAR protists, including apicomplexans and stramenopiles. However, it remains unclear whether these proteins perform similar functions across different species and are therefore functionally interchangeable. To determine whether DDP function is conserved across species, we tested whether *TgDDP* orthologs from the related apicomplexans *Neospora caninum* (*NCLIV_026090; NcDDP*) and *Hammondia hammondi* (*HHA_261410; HhDDP*) could substitute for TgDDP function.

**Fig 7:**
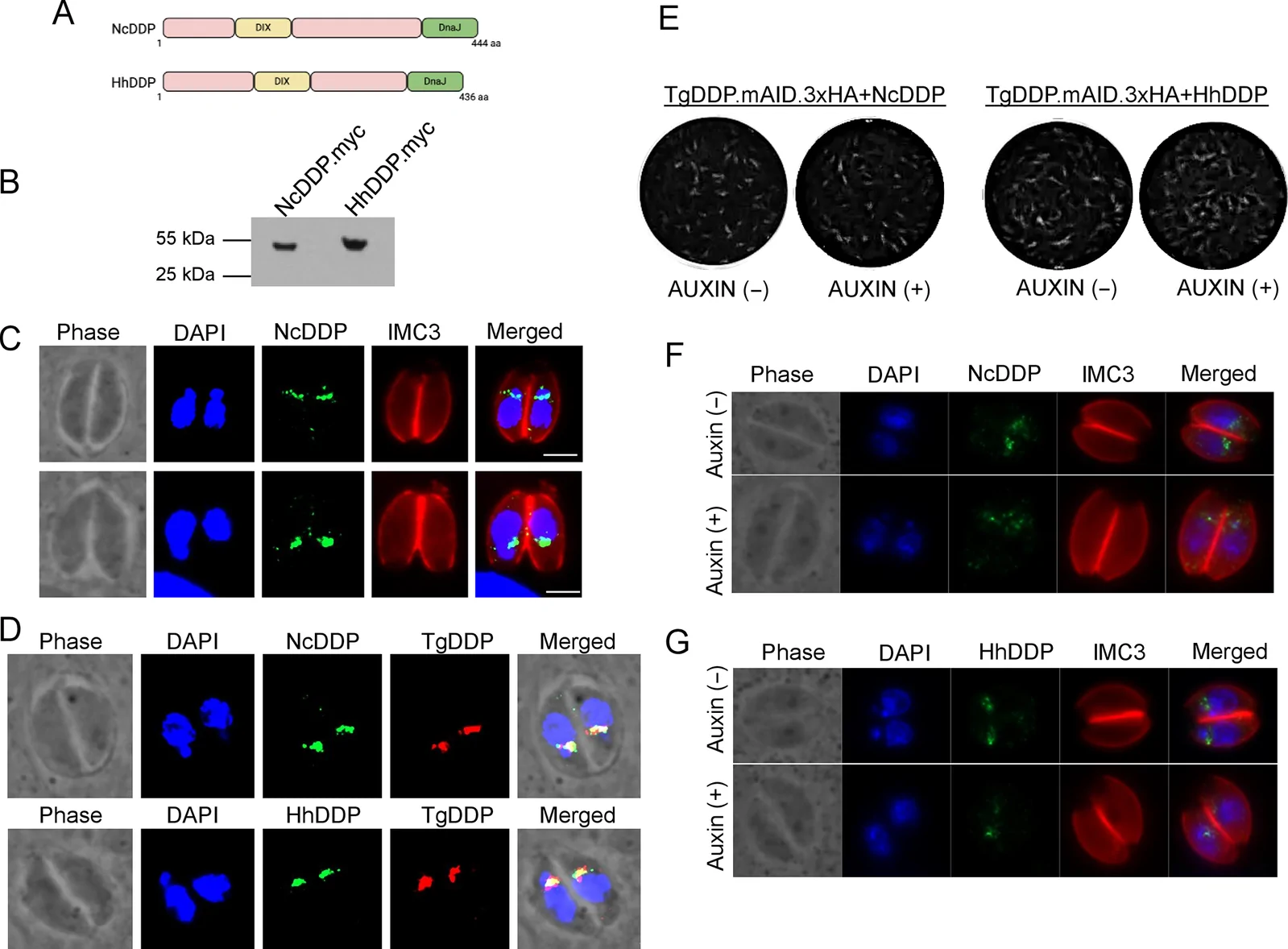
Complementation of the TgDDP.mAID.3×HA strain with orthologous genes rescues the phenotype. (**A**) Schematic representation of NcDDP and HhDDP proteins. (**B**) Immunoblot analysis of TgDDP.mAID.3×HA + NcDDP.myc and TgDDP.mAID.3×HA + HhDDP.myc with anti-myc antibody. (**C**) IFA analysis of NcDDP- and HhDDP-complemented strains using anti-myc antibody and anti-IMC3 antibody. (**D**) IFA analysis of NcDDP- and HhDDP-complemented strains using anti-HA and anti-myc antibodies. (**E**) Plaque assays of TgDDP.mAID.3×HA + NcDDP.myc and TgDDP.mAID.3×HA + HhDDP.myc strains in the presence or absence of auxin. (**F**) IFA of TgDDP.mAID.3×HA + NcDDP.myc strain in the presence or absence of auxin. (**G**) IFA of TgDDP.mAID.3×HA + HhDDP.myc strain in the presence or absence of auxin. DAPI was used to stain the nucleus in all IFAs. Scale bar: 2 μm.

The NcDDP protein consists of 444 amino acids, whereas HhDDP contains 436 amino acids. However, both proteins possess DIX and DnaJ domains similar to those found in TgDDP (Fig. 7A). TgDDP shares 88% sequence identity and 90% sequence similarity with HhDDP, and 65% sequence identity and 74% sequence similarity with NcDDP (Data Set S1). To assess whether these orthologs can substitute for *TgDDP*, we generated plasmid constructs in which the cDNA of each *TgDDP* ortholog, fused to a C-terminal myc tag, was placed under the control of the *TgDDP* promoter and transfected into the TgDDP.mAID.3×HA strain. The constructs were targeted to the *UPRT* locus, and stable parasite clones were obtained using FUDR selection.

Immunoblot analysis of TgDDP.mAID.3×HA + NcDDP.Myс and TgDDP.mAID.3×HA + HhDDP.Myс strains showed a single band at the expected molecular weight for each ortholog (Fig. 7B). Immunofluorescence analysis of the transgenic strains indicated that the orthologous proteins localize to a region between the nucleus and the apicoplast, similar to the localization observed for TgDDP in the parasite (Fig. 7C). To further examine the localization of NcDDP and HhDDP within the parasite, IFAs were performed using anti-HA and anti-myc antibodies. These analyses showed that TgDDP and the orthologous proteins largely colocalize (Fig. 7D), suggesting that NcDDP and HhDDP undergo trafficking similar to TgDDP in *Toxoplasma*.

Next, we sought to determine whether the orthologous proteins can functionally replace TgDDP in the conditional knockdown mutant. To address this, plaque assays were performed using TgDDP.mAID.3×HA + NcDDP.Myс and TgDDP.mAID.3×HA + HhDDP.Myс strains in the presence or absence of auxin. Both strains were able to grow and form plaques under both conditions (Fig. 7E and 3). Further immunofluorescence analysis showed that parasites expressing NcDDP or HhDDP were able to undergo normal division and form daughter cells (Fig. 7F and G). These results indicate that both orthologous proteins can functionally substitute for DDP in *Toxoplasma*.

### TgDDP forms oligomers

Previous studies have shown that DIX domain-containing proteins, such as DVL2 in mammals and SOSEKI proteins in plants, undergo polymerization. Since TgDDP also contains a DIX domain, we sought to determine whether it similarly forms oligomers.

**Fig 8:**
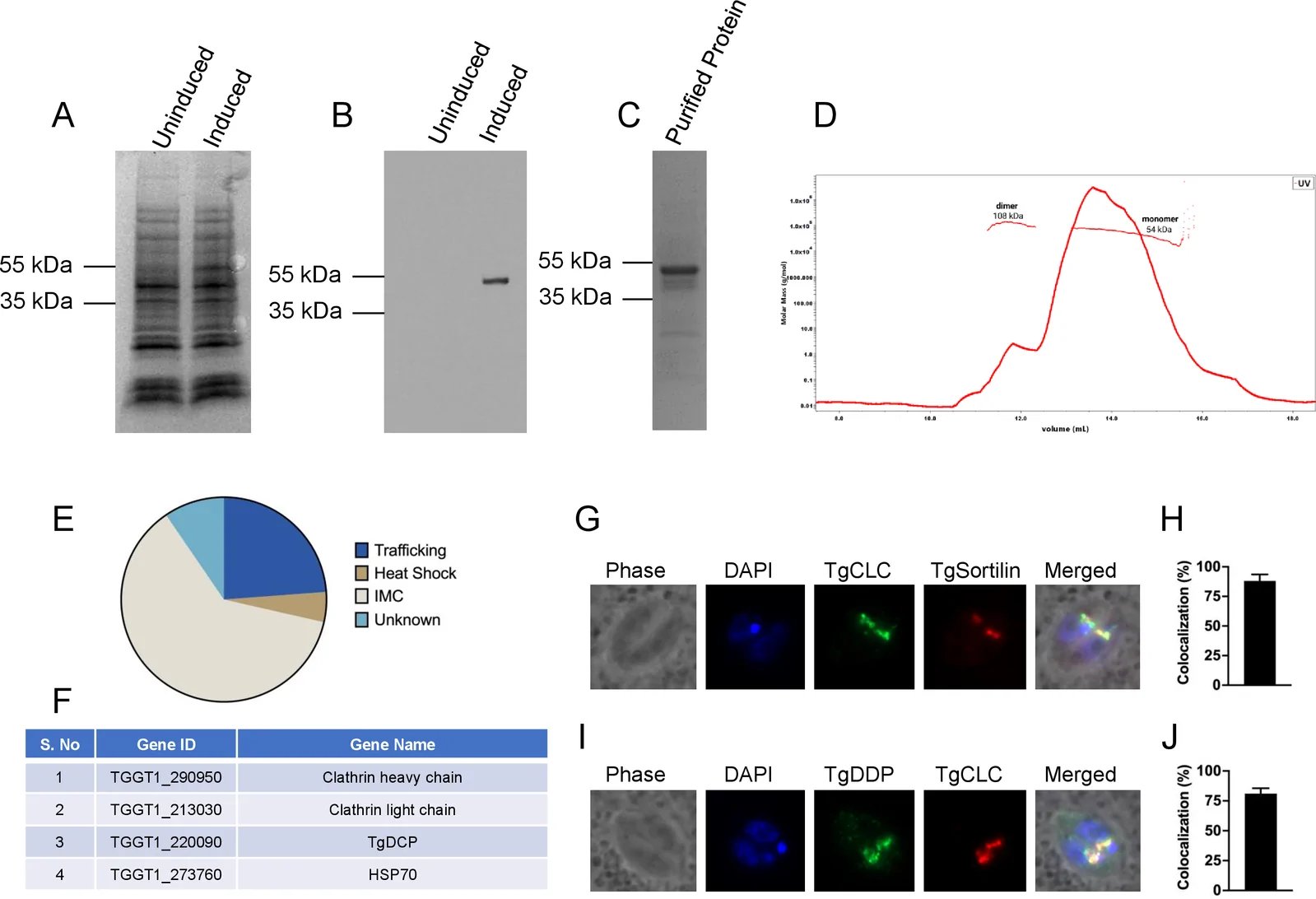
Expression, purification of recombinant TgDDP from *E. coli*, and SEC-MALS analysis (**A–D**), and TgDDP interactome (**E–J**). (**A**) Coomassie blue-stained uninduced and induced bacterial lysate expressing TgDDP. (**B**) Recombinant TgDDP was purified from *E. coli* and verified by immunoblotting using anti-His antibody. (**C**) Coomassie blue-stained purified TgDDP. (**D**) SEC-MALS analysis of 1.5 mg/mL TgDDP in SEC buffer. Molar masses were calculated using measurements taken from the UV detector, laser monitor correction modes, and refractive index signals as a function of elution volume. The molar mass measurement of 10,000 (1.0 × 104) represents 10 kDa. (**E**) Pie chart showing different categories of proteins that interact with TgDDP. (**F**) List of trafficking proteins that were found to interact with TgDDP. (**G–J**). TgCLC shows colocalization with TgDDP. (**G**) IFA analysis of TgDDP.3×HA-TgCLC.3×myc strain using anti-myc and anti-TgSortilin antibodies. (**H**) Quantification of colocalization of Sortilin and TgCLC. (**I**) IFA analysis of TgDDP.3×HA-TgCLC.3×myc strain using anti-myc and anti-HA antibodies. (**J**) Quantification of colocalization of TgDDP and TgCLC.

To investigate this, the *TgDDP* cDNA was amplified and cloned downstream of the lac promoter in the bacterial expression vector pET28+. After sequence validation, the plasmid was transformed into *E. coli*, and recombinant protein expression was induced with IPTG. Induction yielded a protein band of expected size, and the expression of His-tagged TgDDP was confirmed by immunoblotting using an anti-His antibody (Fig. 8A and B). The recombinant protein was purified and verified by Coomassie blue staining (Fig. 8C).

To assess the oligomerization state of TgDDP, size exclusion chromatography coupled with multi-angle light scattering (SEC-MALS) was performed (41). In this method, proteins are first separated based on size and then analyzed to determine their absolute molecular weight in solution. By comparing the measured molecular weight with the theoretical value, SEC-MALS enables precise determination of a protein’s assembly state under native conditions. Using this approach, we found that TgDDP exists not only as a monomer (~54 kDa) but also as a dimer (~108 kDa) (Fig. 8D). SEC-MALS analysis did not reveal higher-order oligomeric species. These findings indicate that TgDDP possesses an intrinsic ability to oligomerize, and its capacity to form dimers could be important for its functional role in the cell.

### Interactome of DDP

Identification of protein–protein interactions can provide important insights into the molecular function of a protein. Therefore, to characterize the DDP interactome, we performed immunoprecipitation coupled with mass spectrometry (IP-MS). More specifically, we performed immunoprecipitation of TgDDP in the epitope-tagged TgDDP.3×HA strain and included the parental strain RH∆Ku80 as the negative control. The IP-MS analysis identified several proteins involved in trafficking, including clathrin heavy chain (TgCHC, TGGT1_290950), clathrin light chain (TgCLC, TGGT1_213030), and TgHsp70 (TGGT1_273760), as well as TgDCP and multiple IMC proteins (Fig. 8E and F; Data Set S2). These findings identify a set of candidate proteins that may associate with TgDDP and potentially contribute to its role in trafficking and daughter cell assembly.

Proteins that interact with each other are often expected to show colocalization within the cell. Therefore, to further qualitatively assess one of the candidate associations identified by IP-MS, we endogenously tagged TgCLC at the C-terminus with a 3×myc epitope in the TgDDP.3×HA strain. IFA analysis of the TgDDP.HA-TgCLC.3×myc strain using anti-Sortilin and anti-myc antibodies showed approximately 88% colocalization of TgCLC with the trans-Golgi network compartment in the parasite (Fig. 8G and H). We further performed IFA analysis of the TgDDP.3×HA-TgCLC.3×myc strain using anti-myc and anti-HA antibodies. The results showed approximately 81% colocalization of TgDDP and TgCLC (Fig. 8I and J). Although these colocalization studies provide additional support for the putative association identified by IP-MS, further biochemical validation will be necessary to confirm these protein–protein interactions.

## DISCUSSION

Daughter cell budding and cytokinesis are essential stages of cell division in the model apicomplexan parasite *Toxoplasma gondii* (4), yet many proteins involved in these processes remain poorly characterized. In this study, we identify TgDDP as a novel member of the DIX domain-containing protein family that is restricted to stramenopiles and apicomplexans. DDP is distinct among known DIX proteins because it contains both a DIX domain and a DnaJ domain within the same polypeptide. Proteins with a similar domain organization have recently been identified in *Phytophthora palmivora*, an oomycete belonging to the SAR supergroup and an important plant pathogen (26).

Our studies demonstrate that DDP predominantly localizes to the parasite TGN, with additional localization to discrete punctate structures in the cytoplasm. During parasite division, DDP exhibits a more diffuse distribution, with a fraction of the protein associating with the IMC of newly forming daughter cells. Conditional depletion of DDP revealed that this protein is essential for parasite replication, as loss of DDP results in a profound defect in daughter cell budding and cytokinesis. Electron microscopy further revealed cellular abnormalities following DDP depletion, including swelling of the nuclear envelope, extensive vacuolization, loss of parasite polarity, and eventual cellular disintegration. In addition, altered association of host ER and mitochondria with the parasitophorous vacuole membrane (PVM) of the dying parasites was observed, although these changes may represent secondary consequences of impaired parasite development.

The identification of multiple trafficking proteins, including clathrins, as well as numerous IMC-associated proteins in the DDP putative interactome supports a model in which DDP contributes to membrane trafficking events required for daughter cell assembly. However, given its localization at the TGN and the cellular abnormalities observed following depletion, it is also feasible that DDP contributes to additional aspects of intracellular membrane organization beyond its role in daughter cell assembly. Whether these effects represent direct functions of DDP or secondary consequences of its absence remains to be determined. The extensive vacuolization observed by electron microscopy following DDP depletion may also reflect cellular stress responses, including potential ER stress, although further studies will be required to distinguish primary effects of DDP loss from downstream consequences of defective parasite division.

Although proteins containing both DIX and DnaJ domains are restricted to stramenopiles and apicomplexans, DDP orthologs display notable species-specific variation. For example, within the *Plasmodium* genus, the highly pathogenic species *Plasmodium falciparum* appears to lack a protein containing both domains. In *Phytophthora* species, DIX domain-containing proteins exhibit four distinct domain architectures and have been classified based on characters from *The Three Musketeers* by Alexandre Dumas (26). Proteins containing only the DIX domain are designated ATHOS, those containing DIX and kinase domains are termed PORTHOS; proteins containing DIX and engulfment and cell motility (ELMO) domains are designated ARAMIS; and proteins containing both DIX and DnaJ domains are classified as DARTAGNAN. Based on this classification, DDP corresponds to the DARTAGNAN family, whereas DCP corresponds to the ATHOS family identified in *Phytophthora* species.

The DIX domain proteins show varied localization in higher eukaryotes. In humans, DIX domain proteins such as Axin and DVL2 localize to the plasma membrane along with characteristic discrete punctae in the cytosol (24). In plants, SOSEKI proteins are the DIX domain proteins, and these predominantly show polar localization in cells (23). However, it is interesting to note that in unicellular organisms such as *Toxoplasma*, the DIX domain-containing DDP predominantly localizes to the trans-Golgi network, although we do see the cytoplasmic puncta in the parasite cytosol. However, the significance of these discrete cytosolic spots of the DIX proteins is yet to be determined.

DDP contains two notable domains, DIX domain and the DnaJ domain. However, the precise roles and essentiality of these two domains to the functioning of DDP are not known and would be an avenue for our future studies. Interestingly, DDP shares some features with Auxilin protein that has been shown to play a role in clathrin coat disassembly (42, 43). Both Auxilin and DDP contain a DnaJ domain toward the C-terminus, and both interact with trafficking proteins, clathrins, and heat shock proteins. However, the N-terminal region of Auxilin is quite distinct from that of DDP, where it has a PTEN domain. It is feasible that PTEN is an evolutionary replacement for the DIX domain and hence that Auxilin and DDP perform similar functions in the opening up of clathrin-coated vesicles. Further studies on this angle will reveal if this hypothesis is indeed true.

The DIX domain is known to mediate protein polymerization and higher-order assembly in several eukaryotic systems (23, 25). Mammalian DVL proteins and plant SOSEKI proteins undergo DIX-dependent polymerization, which is critical for their biological functions. Consistent with this property, we found that TgDDP exists not only as a monomer but also as a dimer. Although our data do not demonstrate canonical DIX-dependent polymerization, it is possible that DIX–DIX domain interactions contribute to TgDDP dimerization, which could be important for its biological function.

Interestingly, our TgDDP interactome identified TgDCP as a putative interactor. This finding is particularly intriguing because TgDCP contains a DIX domain but lacks the C-terminal DnaJ domain present in TgDDP. The identification of TgDCP as a TgDDP-associated protein is consistent with the recent observation that DARTAGNAN and ATHOS proteins can interact *in vivo* in SAR organisms (26). Together, these findings raise the possibility that TgDDP and TgDCP may participate in conserved DIX domain-dependent heteromeric assemblies that play a critical role in *Toxoplasma* division. However, the molecular basis and functional significance of the TgDDP–TgDCP association remain to be determined. Future studies will be required to establish whether this interaction is directly mediated through their DIX domains, whether TgDDP and TgDCP form higher-order assemblies, and how these assemblies contribute to parasite division.

The identification of TgDDP as a TGN-associated protein and its interaction with trafficking factors suggests that DDP may regulate key protein transport pathways essential during endodyogeny. Like DDP, Sortilin is a TGN-resident protein involved in intracellular protein trafficking (33). Conditional knockdown of Sortilin results in defects in the biogenesis of the secretory organelles, including micronemes and rhoptries (33). Similarly, depletion of DDP leads to defects in IMC biogenesis in addition to the mis-localization of micronemes and rhoptries. Notably, disruption of IMC biogenesis has not been reported following Sortilin knockdown, suggesting that DDP may have a broader role in coordinating secretory trafficking with parasite cell division. Consistent with this possibility, loss of DDP appears to cause a more pronounced disruption of parasite architecture than has been reported following Sortilin knockdown. One possible explanation for this difference is the conditional depletion strategy used. Sortilin was previously analyzed using an anhydrotetracycline (ATc)-based conditional knockdown system, which generally results in slower protein depletion than the auxin-inducible degron (AID) system used in the present study. Consequently, it is possible that rapid depletion of Sortilin using an AID-based approach would reveal additional phenotypes, including altered parasite morphology or defects in cell division, that were not apparent with the slower knockdown strategy.

The functional conservation of DDP across apicomplexan species provides additional insight into the importance of this protein family. Introduction of a wild-type copy of *TgDDP* successfully rescued the cell division defect observed following endogenous DDP depletion. Furthermore, orthologs from *Neospora caninum* and *Hammondia hammondi* were able to functionally substitute for TgDDP, indicating that the molecular function of DDP is conserved among related apicomplexans. Both orthologs maintain the characteristic organization of the DIX domain at the N-terminus and the DnaJ domain at the C-terminus, suggesting that conservation of these domains contributes to functional preservation. Consistent with this idea, the DIX and DnaJ domains exhibit higher sequence conservation than the overall protein sequence, suggesting that evolutionary pressure has maintained these regions because of their functional importance.

Collectively, our findings identify DDP as a novel protein that links TGN-associated protein transport with daughter cell formation during *Toxoplasma* endodyogeny. Loss of DDP disrupts IMC biogenesis, daughter cell budding, and cytokinesis, while nuclear duplication proceeds normally, indicating that DDP functions primarily during the mid-to-late stages of parasite division. The localization of DDP at the TGN, its association with clathrin-related trafficking machinery, and its interaction with IMC proteins support a model in which DDP contributes to the trafficking of membrane and protein cargo required for daughter cell assembly. Future studies defining the molecular mechanisms of DDP function, including the contributions of its DIX and DnaJ domains and its interactions with DIX-containing and trafficking partners, will provide further insight into how this protein contributes to the cellular processes underlying daughter cell formation.

## MATERIALS AND METHODS

### Parasite cultures

*Toxoplasma gondii* tachyzoites were maintained by passage through human foreskin fibroblasts (HFFs) in a humidified incubator at 37°C with 5% CO_2_. Normal growth medium consisted of DMEM supplemented with 10% fetal bovine serum, 2 mM L-glutamine, and 50 μg/mL of penicillin-streptomycin. Purification of parasites was performed as previously described (44).

### Phylogenetic tree construction and analysis

For predicting the DIX domain protein in the eukaryotic genome sets, we used a custom set of 127 genomes and their associated protein predictions available from the eggNOG v5.0 database (45) and (NCBI) databases (46). These genomes were chosen because they represent a large breadth of eukaryotic diversity. We then used these eukaryotic genomes to search against the Pfam database version 32.0 (47) as a query using the hmmsearch command in HMMER3 (48) (--cut_nc parameter). Sequences that had hit the DIX domain (accession: PF00778) were then used to reconstruct a phylogenetic tree.

For phylogenetic analysis, we aligned the sequences using Clustal Omega v1.2.4 with default parameters (49). Alignment trimming was performed using trimAl v1.4. Rev15 (6), and 90% of the gaps were removed. We then reconstructed the phylogenetic tree using IQtree v1.6.12 (50) with options -bb 1000 (1000 ultrafast bootstraps [51]), -nt AUTO, -m MFP (ModelFinder to determine the best fit model [52]), and five runs. The JTT + F + R6 model was chosen as the best-fit model by ModelFinder. The tree was then visualized using iTOL (53). To find the other associated domains, a conserved domain search was done using the CDD search tool (54) in NCBI. Domain hits were then represented in visual format along with the phylogenetic tree in iTOL.

### Structural modeling

A structural model of TgDDP was generated using AlphaFold3 (27) via the AlphaFold prediction server. The predicted structure was exported as a PDB file and analyzed using PyMOL v3.0 . Residue-level predicted Local Distance Difference Test (pLDDT) confidence scores were used to assess model reliability and guide structural interpretation. Regions corresponding to the previously annotated DIX domain (residues 120–179) and DnaJ domain (residues 394–437) exhibited comparatively high pLDDT scores relative to the remainder of the protein sequence, indicating increased confidence in the predicted structure within these domains. To independently evaluate predicted secondary structure elements, PSIPRED (28, 29) was used to generate α-helix and β-strand predictions from the TgDDP amino acid sequence. The predicted secondary structure elements showed strong agreement with the AlphaFold3 model within the DIX and DnaJ regions, further supporting the reliability of the modeled domain architectures. FASTA sequences of TgDDP and NcDDP were input into the Eukaryotic Linear Motif database to investigate short linear motifs localized to the intrinsically disordered coils. Structural figures were generated and rendered using ray tracing in PyMOL (55).

### Endogenous tagging of TgDDP with HA and mAID.HA epitopes

The endogenous tagging of TgDDP at the C-terminus was performed according to previously published protocols (31, 32, 35). Briefly, a single guide RNA (sgRNA) plasmid containing a protospacer against the 3′ untranslated region (UTR) of *TgDDP* downstream of the stop codon was generated through site-directed mutagenesis. The homology-directed repair (HDR) templates were PCR-amplified using the vectors, p3XHA.LIC-DHFR and pmAID3xHA.LIC-HPT, that contain 3×HA and mAID.3×HA epitope tags, respectively. Additionally, both the plasmids contain a selection cassette that provides resistance against pyrimethamine (*DHFR*) and MPA-xanthine (*HXGPRT*). The 60 bp primers used to generate the repair template included 40 bp of homology immediately upstream of the stop codon or 40 bp of homology within the 3′ UTR downstream of the CRISPR/Cas9 cut site. All primers used for pU6-Universal plasmids and HDR templates are listed in Table S1. The sgRNA plasmid and the HDR templates were then transfected into the RHΔKu80 strain using a nucleofector (56). Transfected parasites were then cultured in the presence of either pyrimethamine or MPA/xanthine to select stably transformed parasites that were cloned by limiting dilution. The clones were screened and validated by PCR and sequencing.

### Endogenous tagging of TgCLC with myc epitope in the TgDDP.HA strain

The endogenous tagging of *TgCLC* at the C-terminus was performed according to previously published protocols (31, 32, 35). Briefly, a single guide RNA (sgRNA) plasmid containing a protospacer against the 3′ untranslated region (UTR) of TgCLC downstream of the stop codon was generated through site-directed mutagenesis. The homology-directed repair (HDR) templates were PCR-amplified using the vector p3Xmyc.LIC-CAT which contains a myc epitope tag. Additionally, the plasmid contains a selection cassette that provides resistance against chloramphenicol. The 60 bp primers used in generating the repair template include 40 bp of homology immediately upstream of the stop codon or 40 bp of homology within the 3′ UTR downstream of the CRISPR/Cas9 cut site. All primers that were used for pU6-Universal plasmids and HDR templates are listed in Table S1. The sgRNA plasmid and the HDR templates were then transfected into the TgDDP.3×HA strain using a nucleofector (56). Transfected parasites were then cultured in the presence of chloramphenicol to select stably transformed parasites that were cloned by limiting dilution. The clones were screened and validated by PCR and sequencing.

### Immunofluorescence microscopy

Immunofluorescence staining of intracellular parasites was performed according to a previously described procedure (57). The primary antibodies used were as follows: mouse anti-HA (Cell Signaling Technology, 6E2, 1:1000), rabbit anti-HA (Cell Signaling Technology, C29F4, 1:1000), rabbit anti-IMC6 (1:1000), rabbit anti-IMC3 (1:2000), rabbit anti-centrin 1, mouse anti-sortilin (1:500), guinea pig anti-SERCA (1:500), rabbit anti-VP1 (1:1000), rabbit anti-ATRX1 (1:500), rabbit anti-F_0_F_1_ ATPase (1:1000), rabbit anti-M2AP (1:1000), rabbit anti-ROP7 (1:500), and rabbit anti-GRA7 (1:500) (58–60). Secondary antibodies used include Alexa Fluor-594- or Alexa Fluor-488-conjugated goat anti-rabbit or goat anti-mouse (Molecular Probes, 1:1,000). Slides were viewed using a Zeiss Axio Observer 7 microscope (Carl Zeiss), and digital images were captured with an Axiocam 506 mono charge-coupled device camera using Zen software.

### Quantification of colocalization

Colocalization analysis was performed using ZEN imaging software (Carl Zeiss). Fluorescence signals from the Alexa Fluor 488 (green) and Alexa Fluor 594 (red) channels were analyzed within defined regions of interest. Background fluorescence was excluded by applying intensity thresholds to each channel, followed by pixel-by-pixel analysis to identify overlapping fluorescence signals. The colocalization coefficient was calculated and expressed as the percentage of overlapping fluorescence signal relative to the total fluorescence signal within each region of interest. For each experiment, five vacuoles were imaged and analyzed. Quantitative data represent the combined results from three independent experiments.

### Electron microscopy

For ultrastructural observations of infected cells by thin section, samples were fixed in 2.5% glutaraldehyde in 0.1 mM sodium cacodylate and processed as described previously (61). Ultrathin sections of infected cells were stained with osmium tetroxide before examination with Hitachi 7600 EM under 80 kV equipped with a dual AMT CCD camera system.

### Generation of TgDDP-complemented strain

To obtain a TgDDP-complemented parasite cell line, we amplified *TgDDP* cDNA using *Toxoplasma* cDNA as template and cloned into pBS SK(+) vector. We then PCR amplified about 1.5 kb of the TgDDP promoter region and cloned it upstream of the *TgDDP*-cDNA sequence to generate TgDDP(pro)-TgDDPcDNA construct. The homology-directed repair (HDR) template that included the TgDDP-cDNA under the control of the native promoter was amplified by PCR using primers that contain 40 bp homology to the UPRT gene locus. We also generated CRISPR-Cas9 plasmids with sgRNA sequences that would cut the *UPRT* gene using a Q5 Site-Directed Mutagenesis Kit (31, 32). The two sgRNAs that cut the *UPRT* after the start codon and near the stop codon were transfected along with the HDR template, and parasites were cultured in the presence of FUDR to select for the loss of *UPRT*. The parasites were then cloned and validated by PCR and sequencing. The primers used in generating the complemented strain and sgRNA plasmids are listed in Table S1.

### Complementation of TgDDP conditional knockdown mutant with ortholog genes

To generate parasite lines stably expressing ortholog genes in the TgDDP.mAID.3×HA background, we synthesized codon-optimized cDNA copies of *NcDDP* and *HhDDP* cDNA with myc epitope tags at the C-terminus from the commercial manufacturer Integrated DNA Technologies (IDT). The cDNA copies with epitope tags were then cloned downstream of the TgDDP promoter to generate TgDDP.NcDDP.myc and TgDDP.HhDDP.myc constructs. Next, we amplified the homology-directed repair (HDR) template, which included 40 bp homology to the UPRT gene locus on either end, with TgDDP.NcDDP or TgDDP.HhDDP in the center, by PCR. The HDR was then transfected with CRISPR-Cas9 plasmids that would cut the *UPRT* gene into TgDDP.mAID.HA strain, and stable transformants were selected and cloned in the presence of FUDR.

### Plaque assays

Intracellular parasites were harvested, syringe-filtered, and added onto a confluent monolayer of HFF cells in a 12-well plate (500 tachyzoites per well). The plates were then incubated at 37°C for 6 days without any disturbance. The plates were then washed with PBS, methanol fixed, and stained with 2% crystal violet to visualize regions of host cell disruption (56).

### Invasion assay

Invasion assays were performed in eight-well chamber slides as previously described, with the following modifications (62). Purified tachyzoites were added onto HFF monolayers (2 × 10^6^ parasites per well) and incubated at 37°C for 30 min. Slides were then washed three times to remove non-invaded parasites, fixed, blocked, and stained with mouse anti-SAG1 without permeabilization. After 1 h, slides were washed, permeabilized with 0.01% TX-100, and stained with rabbit anti-M2AP antibody. The slides were further washed and stained with secondary antibodies, Alexa Fluor-594-conjugated goat anti-mouse (Molecular Probes) and Alexa Fluor-488-conjugated goat anti-rabbit (Molecular Probes). After 1 h, slides were washed and mounted using Vectashield (with DAPI). Parasites that were both red and green were identified as extracellular (attached), whereas those that were green but not red were identified as intracellular (invaded). Images of 15 random fields of view within each well were captured at 600× magnification, and the total number of intracellular parasites and host-cell nuclei were enumerated.

### Replication assay

To assess the parasite doubling time, freshly egressed parasites were inoculated into confluent HFF monolayers in 12-well plates and allowed to invade for 2 h. The monolayers were then washed three times with medium to remove uninvaded parasites and incubated at 37°C. At 24 h post-infection, the cells were fixed with methanol and stained using Diff-Quik (Dade-Behring) according to the manufacturer’s instructions. For each treatment, at least 100 vacuoles from three biological replicates were assessed for the number of parasites per vacuole (56).

### Ionophore-induced egress assay

The efficiency of egress after calcium ionophore treatment was determined using established protocols (38). Briefly, freshly harvested parasites were added to a 24-well plate containing confluent HFFs at a multiplicity of infection (MOI) of 1 and were incubated at 37°C for 30 h. To induce egress, intracellular parasites were washed with warm PBS, incubated at 37°C for 2 min in Hanks’ balanced salt solution (HBSS) containing 1 μM calcium ionophore A23187, and fixed with 100% methanol. To visualize intact and lysed vacuoles, the cultures were stained using Diff-Quik (Dade-Behring) according to the manufacturer’s instructions. Percent egress was determined by dividing the number of lysed vacuoles by the total number of vacuoles in a sample.

### Expression and purification of recombinant TgDDP

The ORF coding for *TgDDP* was amplified from parasite cDNA using PCR and cloned into the *E. coli* expression vector pET28a + in frame with a C-terminal His tag, and the plasmid was transformed into Rosetta DE3 cells. The DE3 cells were grown at 37°C in LB containing 100 μg/mL kanamycin and chloramphenicol to an OD_600_ of 0.6, and gene expression was induced with 1 mM IPTG (isopropyl-β-D-1-thiogalactopyranoside). After 5 h, growth cells were harvested by centrifugation, and the native recombinant protein was purified using QIAexpress Ni-NTA Fast Start Kit (Qiagen) according to the manufacturer’s instructions. Protein elution was monitored by UV absorbance at a wavelength of 280 nm and confirmed by SDS-PAGE. Pooled fractions containing recombinant protein were further purified by size-exclusion chromatography using a HiPrep 26/60 Sephacryl S-200 HR column (GE Healthcare) in 100 mM NaCl and 50 mM HEPES, pH 7.4, at a flow rate of 1 mL/min. Protein-containing elution fractions were pooled and concentrated using an Amicon ultrafiltration system and 10 kDa regenerated cellulose ultrafiltration discs (Millipore, Billerica, MA) for SEC-MALS experiments.

### Size exclusion chromatography and multiangle light scattering (SEC-MALS)

The molecular mass of TgDDP was determined by size exclusion chromatography with online absorbance, multiangle light scattering (MiniDawn, Wyatt Technology), and refractive index detectors (Optilab). Purified protein at a concentration of 1.5 mg/mL was incubated at room temperature for 30 min before being injected into a Superdex 200 increase (10/300 GL) column (Cytiva), which was equilibrated in 100 mM NaCl and 50 mM HEPES at 22°C and a flow rate of 0.5 mL/min. The refractive index (RI) signal was used as a concentration source for analyzing the light scattering data with the ASTRA program (version 8.1.2.1; Wyatt Technology).

### Immunoprecipitation-mass spectrometry (IP-MS)

The immunoprecipitation of TgDDP was performed using epitope-tagged TgDDP.3×HA strain, and the parental strain RH∆Ku80 was used as the negative control. Intracellular parasites were harvested by scraping the HFF monolayer, followed by syringe filtration and centrifugation. The parasites were then washed with PBS and treated with Pierce lysis buffer. The samples were then sonicated, centrifuged, and the supernatant fraction containing soluble proteins was obtained. The soluble protein complex was then incubated with washed Pierce anti-HA magnetic beads. The protein fraction unbound to the magnetic beads was removed using a magnetic stand, and the beads were washed three times before resuspension in PBS. The samples were then submitted to Virginia Tech Mass Spec Incubator Facility. The mass spectrometric analysis was carried out on a Thermo Fisher Scientific LTQ Orbitrap Velos Pro mass spectrometer (Thermo Fisher Scientific, Waltham, MA) interfaced with a Waters Acquity UPLC system (Waters, Milford, MA). The proteins bound to magnetic beads were directly digested by trypsin. Samples were first reduced with 10 mM DTT in 10 mM ammonium bicarbonate and then alkylated with 55 mM iodoacetamide (prepared freshly in 10 mM ammonium bicarbonate). Alkylated samples were digested by trypsin overnight at 37°C. Tryptic peptides were first injected onto a C18 trapping column (NanoAcquity UPLC Trap column 180 μm × 20 mm, 5 μm, Symmetry C18) and subsequently onto an analytical column (NanoAcquity UPLC column 100 μm × 100 mm, 1.7 μm BEH130 C18). Peptides were eluted with a linear gradient from 3% to 40% acetonitrile in water containing 0.1% formic acid over 90 min at room temperature at a flow rate of 500 nL/min, and the effluent was electrosprayed into the LTQ Orbitrap mass spectrometer. Blanks were run prior to the sample to make sure there were no significant background signals from solvents or the columns. A database search against annotated proteins from the *Toxoplasma gondii* GT1 strain in ToxoDB was performed using Sequest (Thermo Fisher Scientific) search engine to identify proteins.

## Data Availability

The proteome data generated in this study has been deposited to the proteomics repository Mass Spectrometry Interactive Virtual Environment (MassIVE), and the accession number is MSV000103075.

## SUPPLEMENTAL MATERIAL

Data Set S1 mbio.01952-26-s0001.tiff Sequence alignment.

Data Set S2 mbio.01952-26-s0002.xlsx IP data.

Figure S1 mbio.01952-26-s0003.tiff DDP shows dynamic localization during *Toxoplasma* endodyogeny.

Figure S2 mbio.01952-26-s0004.tiff Complementation with wild-type copy of *TgDDP* rescues phenotype in TgDDP.mAID.3×HA strain.

Figure S3 mbio.01952-26-s0005.tiff Plaque numbers of TgDDP.mAID.3×HA strain complemented with either *NcDDP* or *HhDDP* in the presence or absence of auxin.

Legends mbio.01952-26-s0006.docx Supplemental material legends.

Table S1 mbio.01952-26-s0007.docx Primers.

ASM does not own the copyrights to Supplemental Material that may be linked to, or accessed through, an article. The authors have granted ASM a non-exclusive, world-wide license to publish the Supplemental Material files. Please contact the corresponding author directly for reuse.
