# Supplementary material for: TgDDP is a novel DIX domain protein that plays an essential role in Toxoplasma endodyogeny
Source: mBio. 2026 Sep 28;17(11):e01952-26. doi: 10.1128/mbio.01952-26 (PMC13753751; doi:10.1128/mbio.01952-26)
Supplement: Supplementary file 1 [file mbio.01952-26-s0006.docx]

**Legends for supplemental material**

**LEGENDS FOR SUPPLEMENTAL FIGURES**

**Figure S1.** DDP shows dynamic localization during *Toxoplasma* endodyogeny. **A.** IFA analysis of TgDDP.3xHA in different stages of endodyogeny using anti-HA and anti-IMC3 antibodies. DAPI stains the nucleus. The stages of the cell cycle are indicated as G1, early M/C (mitosis/cytokinesis) and late M/C. Scale bar, 2 μm. **B.** Quantification of DDP staining pattern in parasitophorous vacuoles across different cell cycle stages. Data are presented as mean ± standard deviations from three independent experiments.

**Figure S2.** Complementation with wild-type copy of *TgDDP* rescues phenotype in TgDDP.mAID.3xHA strain. **A.** Plaque assays of TgDDP.mAID.3xHA in presence or absence of auxin. **B.** Quantification of plaque numbers. n = 3, Error bars = SD, Student’s *t* test. *P < 0.05. **C.** Plaque assays of TgDDP.mAID.3xHA complemented with wildtype copy of *TgDDP* in presence or absence of auxin. **D.** Quantification of plaque numbers. n = 3, Error bars = SD, Student’s *t* test. ns = not significant. **E.** IFA of TgDDP.mAID.3xHA and complemented strains with or without auxin using antibodies against HA epitope and IMC3. DAPI was used to stain the nucleus. Scale bar, 2 μm.

**Figure S3.** Quantification of plaque numbers of TgDDP.mAID.3xHA strain complemented with either *NcDDP* (**A**) or *HhDDP* (**B**) in presence or absence of auxin. n = 3, Error bars = SD, Student’s *t* test. ns = not significant.

**SUPPLEMENTAL DATA LEGENDS**

1. Supplemental **table S1**. List of primers used in this study.

2. Supplemental **data set S1**. Alignment of TgDDP, NcDDP and HhDDP protein sequences.

3. Supplemental **data set S2**. List of interactors of TgDDP identified through IP-MS approach.
