# Supplementary material for: TgDDP is a novel DIX domain protein that plays an essential role in Toxoplasma endodyogeny
Source: mBio. 2026 Sep 28;17(11):e01952-26. doi: 10.1128/mbio.01952-26 (PMC13753751; doi:10.1128/mbio.01952-26)
Supplement: Supplementary file 2 [file mbio.01952-26-s0007.docx]

Table S1. Primers used in this study.

| S. No | Name | Sequence |
| --- | --- | --- |
| 1 | TgDDP.3’.sg.F | gtcctctctcGTTTTAGAGCTAGAAATAGC |
| 2 | TgDDP.3’.sg.R | cagccaatttAACTTGACATCCCCATTTAC |
| 3 | TgDDP.HA.RT.F | TTTCCAGGCTTTCAACGAGGCCTTCAAAGTCCACCCTCCTggaagtggaggacgggaatt |
| 4 | TgDDP.HA.RT.R | CAGAGAGCTAACACTACGCTGACGTGAAGTAGAGTTGCCGACGGCCAGTGAATTGTAATA |
| 5 | TgDDP.mAID.HA.RT.F | TTTCCAGGCTTTCAACGAGGCCTTCAAAGTCCACCCTCCTgagaagagcgcgtgtcctaa |
| 6 | TgDDP.mAID.HA.RT.R | CAGAGAGCTAACACTACGCTGACGTGAAGTAGAGTTGCcgctctagactgcagataac |
| 7 | TgUPRT.sg1.F | aggcgtgagtGTTTTAGAGCTAGAAATAGC |
| 8 | TgUPRT.sg1.R | gtttccctctAACTTGACATCCCCATTTAC |
| 9 | TgUPRT.sg2.F | acatgccaatGTTTTAGAGCTAGAAATAGC |
| 10 | TgUPRT.sg2.R | cacttcatagAACTTGACATCCCCATTTAC |
| 11 | TgUPRT.DDP.RT.F | GCGATTCCCGTATTGGTCACGCATCCACAAGGCGTATTCCAGGAGAATCGCGGTCCAGAA |
| 12 | TgUPRT.DDP.RT.F | TGGTGTTTATCCTCTTGAGGCGTGCTTTTTCCAGTCCGCGTCAAGGAGGGTGGACTTTGA |
| 13 | TgCLC.SG1.F | gcgaggcctgGTTTTAGAGCTAGAAATAGC |
| 14 | TgCLC.SG1.R | aaccaccagtAACTTGACATCCCCATTTAC |
| 15 | TgCLC.myc.CAT.RT.F | CGTCGAGTTGAGCGCCCAAGAGAAGCCTGGAGAGTCCAACggaagtggaggacgggaatt |
| 16 | TgCLC.myc.CAT.RT.R | ACCGAGTGTCCCACAGTGTCTGTTTCGCGTTCCCTGACAGCGACGGCCAGTGAATTGTAA |
